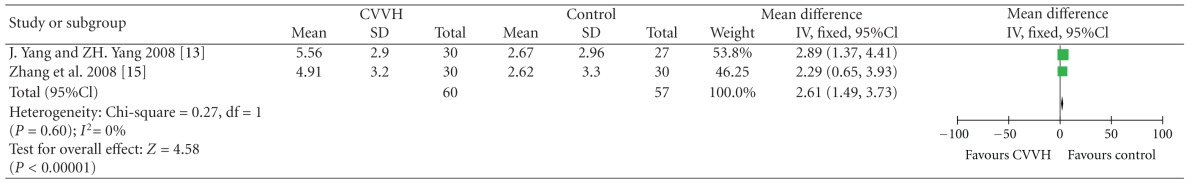# The Effects of Continuous Blood Purification for SIRS/MODS Patients: A Systematic Review and Meta-Analysis of Randomized Controlled Trials

**DOI:** 10.5402/2012/986795

**Published:** 2012-09-26

**Authors:** Tian Hongliang, Zeng Rong, Wang Xiaojing, Sun Rao, Li Lun, Tian Jinhui, Cao Nong, Yang Kehu

**Affiliations:** ^1^Evidence-Based Medicine Center, School of Basic Medical Sciences, Lanzhou University, Lanzhou 730000, China; ^2^The Second Hospital of Lanzhou University, Lanzhou 730000, China; ^3^The First Hospital of Lanzhou University, Lanzhou 730000, China; ^4^Peking Union Medical College Hospital, Beijing 100730, China

## Abstract

*Background*. Continuous veno-venous hemofiltration (CVVH) has aroused great concern in recent years because its effect on clearing inflammatory mediators and its mechanism of clinical effects in the treatment of critical illness has also become a research direction. *Objective*. To evaluate the efficacy of continuous blood purification for systemic inflammatory response syndrome (SIRS)/multiple organ dysfunction syndrome (MODS) patients. *Methods*. A systematic review of the literature was undertaken to assess randomized controlled trials on CVVH. *Results*. 11 RCTs involving a total of 414 patients were included. Compared with the control group, CVVH for SIRS/MODS patients has several advantages including better effects on clearing the plasma inflammatory mediators IL-6 [SMD_3d_ = −0.45, 95%CI, (−0.83, −0.07), SMD_7d_ = −1.07, 95%CI, (−1.52, −0.62)], on plasma TNF-alfa [SMD_3d_ = −0.87, 95%CI, (−1.69, −0.04), SMD_7d_ = −1.42, 95%CI, (−2.49, −0.35)], lower white blood cell (WBC) count [MD = 2.61, 95%CI, (1.49, 3.73)], shorter hospital stays [MD = −7.21 days, 95%CI, (−10.68, −3.74)] and better stability of hemodynamics. However, there is no significant difference in the mortality rate [MODS:RR = 0.62, 95%CI, (0.38, 1.01), SIRS:RR = 0.75, 95%CI, (0.57, 1.08)]. *Conclusions*. The study showed that CVVH was able to eliminate inflammatory mediators (TNF-alfa, IL-6) in plasma effectively, lower WBC count and shorter hospital stays than conventional therapeutic measures.

## 1. Introduction

Continuous blood purification (CBP) has now been extensively employed for the management of systemic inflammatory response syndrome (SIRS), and even multiple-organ dysfunction syndrome (MODS) in critically ill patients [1]. To those patients, some treatments had been put forward targeting on them like monoclonal antibodies (such as anti-TNF antibody), receptor antagonists (such as anti-PAF), and soluble receptors (e.g., TNFR), yet none of these can prevent the release of inflammatory mediators satisfactorily nor remove existing inflammatory mediators, especially TNF-alfa, as a larger molecular weight trimer (54 kd) has become a difficulty of internal clearance. Hemofiltration is commonly used in an intensive care unit setting which is also called continuous venovenous hemofiltration (CVVH) or continuous renal replacement therapy (CRRT). One of the important mechanisms of hemofiltration is that it may play a role in removed inflammatory mediators [2]. Amongst the mediators, the proinflammatory cytokines tumor necrosis factor (TNF-alfa) and interleukin-6 (IL-6) are thought to occupy a key position in the chain of events leading to shock. 

Some trials support this view, but at the same time, some trials say there is no significant evidence found in the relationship between hemofiltration and inflammatory mediators [3, 4]. So there is still controversy regarding this problem. 

The objective of this study is to evaluate the outcomes of CVVH versus control group for SIRS/MODS patients. Moreover, we investigated whether the use of CVVH would result in an improvement of mortality, total duration of hospital stays, white blood cell (WBC) count, and hemodynamic stability in patients, respectively.

## 2. Methods 

The Preferred Reporting Items for Systematic reviews and Meta-Analyses (PRISMA) was used to conduct data extraction.

### 2.1. Study Selection

We searched electronic databases from PubMed (1966–2011.10), the Cochrane Library (Issue 4, 2011), EMBASE (1974–2011.10), Science Citation Index (1974–2011.10), the China Journal Fulltext Database (1994–2011.10), Chinese Scientific Journals Fulltext Database (1989–2011.10), Chinese Biomedical Literature Database (1978–2011.10), with the terms (continuous' venovenous hemofiltration), (continuous renal replacement therapy), (continuous arteriovenous hemofitration), (continuous blood purification), in combination with the medical subject headings. Relevant articles referenced in these publications were downloaded from the databases. The related article function also was used to widen the search results. All abstracts, comparative studies, nonrandomized trials, and citations scanned were searched comprehensively. We also hand-searched the reference lists of every primary study for additional publications. Further searches were done by reviewing abstract booklets and review articles. Trials were included irrespective of the language in which they were reported. 

### 2.2. Data Extraction

Each study was reviewed by 2 researchers for reliability of our meta-analysis. Only randomized controlled trials on CVVH versus conventional therapeutic measures for inflammatory mediators removal were included in the meta-analysis. Two researchers extracted data separately and if there was any controversial, it was confirmed by the third researcher.

### 2.3. Inclusion Criterion

The inclusion criteria for this analysis were randomized controlled trials that compared CVVH with conventional therapeutic measures about patients with critically inflammatory states (systemic inflammatory response syndrome or multiple-organ dysfunction syndrome) [5].

### 2.4. Exclusion Criteria

Trials were excluded if included patients were pregnant, younger than 18 years, in a moribund state, in chronic renal failure, or receiving immunosuppressive therapy.

### 2.5. Statistical Analysis

We summarized available data from all trials reporting results. Computing pooled risk ratios (RR) and their respective 95% confidence intervals by means of a fixed-effects meta-analysis model. For continuous data, the mean difference (MD) is recommended when all trials use the same scale to report their outcomes, while standardized mean difference (SMD) is more appropriate when trials use different scales to report their outcomes, just as data about effects on clearing the plasma inflammatory mediators in the following. All statistical analysis was performed with Review Manager (version 5.1). We used the chi^2^ statistic to assess heterogeneity between trials and the *I* ^2^ statistic to assess the extent of inconsistency. Subgroup analysis was intended to explore important clinical differences among trials that might be expected to alter the magnitude of treatment effect. 

## 3. Results

**Figure 1:**
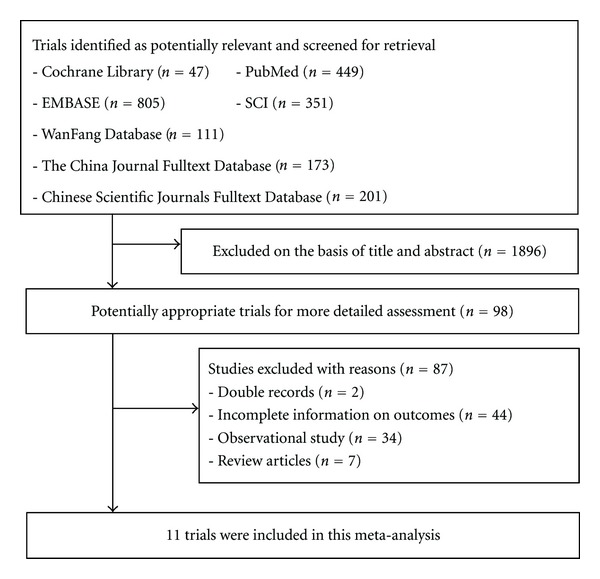
Flow diagram of trial selection.

From Figure 1 can be seen the flow chart of studies from initial results of publication searches to final inclusion [6–16]. 11 trials about CVVH versus conventional therapeutic evaluate the efficacy of inflammatory mediator removals encompassing a total of 438 patients which were retrieved from the electronic databases. Standard deviations were not reported in the majority of studies, where necessarily were estimated either by means of ranges or *P* values. Characteristics of each trial were given in Table 1. Included studies' methodological quality was assessed using the Cochrane handbook in Table 2 [17].

### 3.1. Mortality

Statistical heterogeneity was basically well among studies (*P* = 0.25, *I* ^2^ = 22%) [6–14, 16]. There was no significant difference in mortality between CVVH and Control group (MODS : RR = 0.62, 95%CI, (0.38, 1.01), SIRS : RR = 0.75, 95%CI, (0.57, 1.08)) (Table 3). 

### 3.2. Hospital Stay

Two studies reported the data of length of hospital stay [11, 14]. CVVH group was associated with significantly shorter hospital stays (MD = −7.21 days, 95%CI, (−10.68, −3.74)). Study Cole et al. [9] reported CVVH group shorten the length of stay in the ICU from a Kaplan-Meier analysis (Table 4).

### 3.3. CVVH versus Control on Plasma IL-6 Change

Three studies [10, 11, 15] reported at three-day and seven day followup, CVVH group had better effects than Control group on plasma IL-6 change [SMD_3d_ = −0.45, 95%CI, (−0.83, −0.07), SMD_7d_ = −1.07, 95%CI, (−1.52, −0.62)] (Table 5).

### 3.4. CVVH versus Control Group on Plasma TNF-Alfa Change

There was three trials [10, 11, 15] reported, CVVH versus control on plasma TNF-alfa, significant heterogeneity existed among trials (*P* = 0.07, *I* ^2^ = 69%), (*P* = 0.01, *I* ^2^ = 84%). CVVH group have better effects than Control group on plasma TNF-alfa change (SMD_3d_ = −0.87, 95%CI, (−1.69, −0.04)), (SMD_3d_ = −1.42, 95%CI, (−2.49, −0.35)) (Table 6).

### 3.5. WBC Count Reduction

WBC count reported in two trials was significantly reduced in CVVH group (MD = 2.61, 95%CI, (1.49, 3.73)) [13, 15] (Table 7). 

### 3.6. Hemodynamic Variables

Two trials reported hemodynamic descriptive analysis stability [6, 7]. Riera et al. 1997 [7] reported that there is a significant improvement in mean arterial pressure (80 ± 9 to 94 ± 8 mmHg, *P* = 0.01) and partial pressure of oxygen in arteria blood/inspiratory oxygen supply index (124 ± 40 to 204 ± 44, *P* = 0.03) in the intervention group during the study period. Sander et al. 1997 [6] reported the mean arterial pressure and systemic vascular resistance index tended to increase in the hemofiltration group, but not in the control group. 

## 4. Discussion

 The important effect of inflammatory mediators in the development of SIRS and MODS is recognized. As one of the earliest released inflammatory mediators, TNF-alfa can activate a large amount of inflammatory mediators released by objects like monocyte-macrophage cells and form progressively larger “waterfall-like” chain reaction. TNF-alfa, IL-6, and its mediated cascade play a key role in the pathogenesis of SIRS and MODS [18, 19].

Results for meta-analysis showed that CVVH was able to eliminate inflammatory mediators (TNF-alfa, IL-6) in plasma effectively, lower WBC count, shorten hospital stays, and better stabilize hemodynamics to a greater extent than conventional therapeutic measures. Meanwhile there is no significant difference in mortality between the two groups.

As we know, TNF-alfa and IL-6 affect a wide variety of cells to induce many similar inflammatory reactions: fever, production of cytokines, endothelial gene regulation, chemotaxis, leukocyte adherence, and activation of fibroblasts. They are responsible for the systemic effects of inflammation, such as loss of appetite and increased heart rate. Meta-analysis also reports inflammatory mediators (TNF-alfa, IL-6) and WBC count is significantly reduced in CVVH group. Removal of inflammatory mediators, decrease of inflammatory mediators' body concentration, and keeping the balance of body proinflammatory system and anti-inflammatory system have become ideal therapeutic strategies for those diseases. CVVH treatment in the early stage of MODS patients can decrease or break partly stop the release of cytokines and can improve prognosis [6]. However, Payen et al. study suggests that early application of standard continuous venovenous hemofiltration is deleterious in severe sepsis and septic shock [16].

Nine trials reported the mortality rate, the results of meta-analysis show that there are no significant differences between the two groups [6–14, 16]. Some of the trials was discontinued after There are some reasons like clinical heterogeneity, trial was discontinued after many patients died, limited sample size and limited effect of CVVH can explain it. However, this is a very critical outcome and should be studied carefully in following trials.

 Length of hospital stay results from the studies included in this meta-analysis showed that CVVH significantly reduced length of hospital stay as compared with the control group by 5.3 days. The results are due to the benefits of removal of inflammatory mediators. Reducing length of stay by this amount for every patient is likely to make a significant difference to cost of patient care.

Two studies reported hemodynamic stability [6, 7]. During 48-hour followup, CVVH could keep hemodynamic stability. However, to those SIRS/MODS patients, long-term hemodynamic observation is needed. 

All of the included studies offered adequate descriptions of the randomization process. Only two studies offered allocation concealment [7, 9]. Although blinding of patients and caregivers may not be feasible in CRRT studies, allocation concealment and blinding of data collectors and outcome assessors are possible and desirable. However, blinding, in all of the trials, was not stated, which would yield selection bias and performance bias. Furthermore, only two studies reported intention-to-treat analysis, which would yield attrition bias [9, 16]. All studies reported incomplete outcome data. Due to these methodological limitations, as well as the statistical imprecision and heterogeneity, the quality of evidence presented in this article is considered of lesser quality.

Our meta-analysis also had its limitations. First, there was inability to assess and estimate effects of baseline patient characteristics because access to individual patient data was limited. Second, different types of filter membranes used may have had a different impact. The characteristics of the membrane used in CVVH may be an important factor on cytokine clearance. The use of different membranes may lead to variable TNF and IL-6 extraction. Third, all of the included studies focused on only two inflammatory mediators, (TNF-alfa, IL-6). Perhaps other mediators like IL-1, IL-8, platelet activating factor (PAF), and so forth, can be tested. Fourth, some data should be assessed using weighted mean differences, or only the descriptive analysis can be used. Finally, some of the inclusion studies were from China, so the results should be considered carefully when they were applied to other countries. Therefore, we still need more high-quality, multicenter, randomized, and controlled trials from other countries and regions.

## 5. Conclusions

 All in all, CVVH could eliminate inflammatory mediators (TNF-alfa, IL-6) in plasma effectively, shorten hospital stays, and better stabilize of hemodynamics. This is worthy of further exploration and promotion. As an exogenous way of clearance, CVVH has aroused great concern because its effect on clearing inflammatory mediators and its mechanism of clinical effects in the treatment of critical illness will also become of more and more perspective in research and application.

## Figures and Tables

**Table 1:** Baseline characteristics of the included studies.

Trials	Sample size (*n*)	Country	Disease	Illness severity scores	Age	Filter membrane	Blood flow (mL/min)	Treatment
Sander 1997 [6]	13/13	Germany	SIRS	15.3 ± 1.4/13.9 ± 1.2^A^	58 ± 3.0/52 ± 3.2	AN69	150 mL/min	CVVH* versus control
Sanchez Riera 1997 [7, 8]	15/15	Spain	MODS	22 ± 7/21 ± 6^A^	36 ± 18/36 ± 14	AN69	130 mL/min	CVVH* versus control
Cole et al. 2002 [9]	12/12	Australia	MODS	21.8 ± 4/22.2 ± 6.4^A^	Median (IQR) 65.5 (22.0)/ 68.0 (19.0)	AN69	200 mL/min	CVVH* versus control
Liu et al. 2003 [10]	24/26	China	MODS	25 ± 7/23 ± 7^A^	39 ± 11/37 ± 19	AN69 +PS	200–250 mL/min	CVVH* versus control
Yang et al. 2004 [11]	22/15	China	SIRS	14.8 ± 4.5/14.6 ± 4.7^A^	44.6 ± 15.4/44.6 ± 15.4	PS	150–200 mL/min	CVVH* versus control
Ding and Zhao 2007 [12]	11/10	China	MODS	14.1 ± 3.6/14.1 ± 3.6^A^	23–78/23–78	PS	Not reported	CVVH* versus control
Yang and Hoyang 2008 [13]	31/27	China	SIRS	18.6 ± 3.4/17.9 ± 2.7^A^	31.5 ± 10.9/31.5 ± 10.9	PS	180–250 mL/min	CVVH* versus control
Peng et al. 2008 [14]	17/15	China	SIRS	18.21 ± 2.58/17.65 ± 3.14^A^	28–70/30–69	AN69	250–300 mL/min	CVVH* versus control
Zhang et al. 2006 [15]	30/30	China	SIRS	Not reported	46.7 ± 18.3/46.7 ± 18.3	PS	150–200 mL/min	CVVH* versus control
Payen et al. 2009 [16]	37/39	France	SIRS	11.6 ± 3.4/10.4 ± 2.9^B^	57.6 ± 12.6/58.6 ± 13.5	PS	150 mL/min	CVVH* versus control

MODS: multiple-organ dysfunction syndrome.

SIRS: systemic inflammatory response syndrome.

CVVH* group: CVVH + conventional therapeutic measures.

Control group: conventional therapeutic measures.

A: acute physiology and chronic health evaluation, APACHE II score; B: simplified acute physiology score, SOFA score.

**Table 2:** Methodological quality of included studies.

Trials	Randomization	Allocation concealment	Blinding	Incomplete outcome data	ITT analysis
Sander	Yes	Not stated	Non blind	Yes	Not stated
Sanchez Riera	Yes, random schedule	Yes	Not stated	Yes	Not stated
Cole	Yes, random number table	Opaque-sealed envelopment	Not stated	Yes	Yes
Liu	Yes	Not stated	Not stated	Yes	Not stated
Yang	Yes	Not stated	Not stated	Yes	Not stated
Ding	Yes	Not stated	Not stated	Yes	Not stated
Yang	Yes	Not stated	Not stated	Yes	Not stated
Peng	Yes	Not stated	Not stated	Yes	Not stated
Zhang	Yes, random number table	Not stated	Not stated	Yes	Not stated
Payen	Yes, by blocks of four	Not stated	Not stated	Yes	Yes

**Table 3:** Mortality.

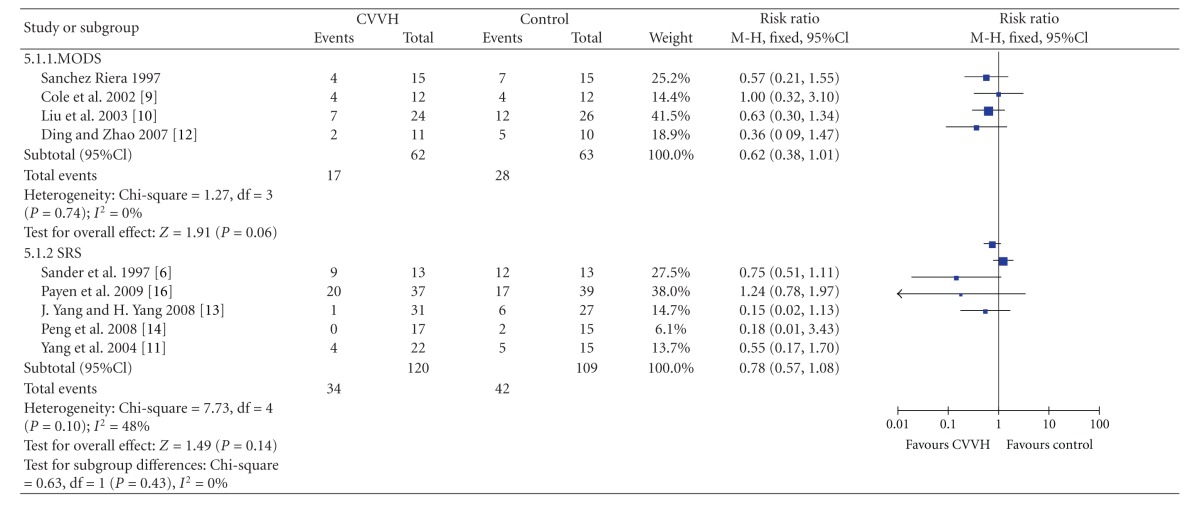

**Table 4:** Hospital stay.

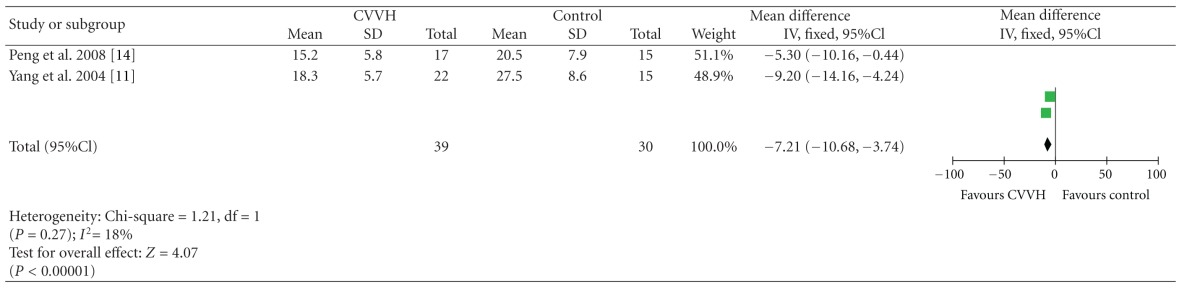

**Table 5:** IL-6 change.

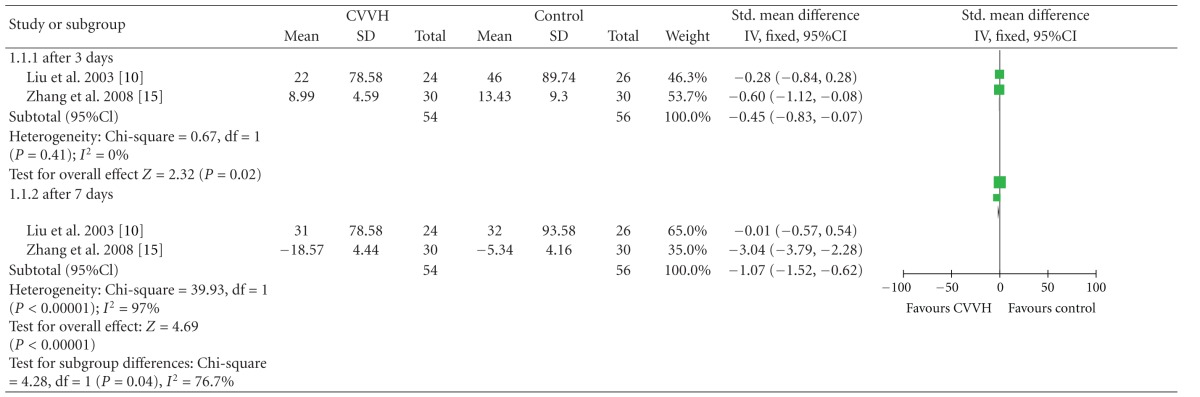

**Table 6:** TNF-*α* change.

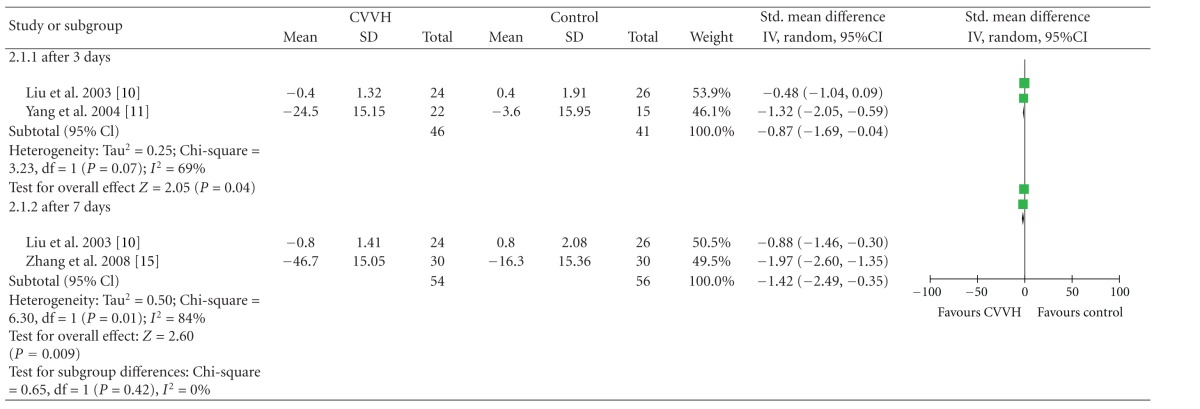

**Table 7:** WBC count reduction.